# Probing spin correlations using angle-resolved photoemission in a coupled metallic/Mott insulator system

**DOI:** 10.1126/sciadv.aaz0611

**Published:** 2020-02-07

**Authors:** V. Sunko, F. Mazzola, S. Kitamura, S. Khim, P. Kushwaha, O. J. Clark, M. D. Watson, I. Marković, D. Biswas, L. Pourovskii, T. K. Kim, T.-L. Lee, P. K. Thakur, H. Rosner, A. Georges, R. Moessner, T. Oka, A. P. Mackenzie, P. D. C. King

**Affiliations:** 1 SUPA, School of Physics and Astronomy, University of St Andrews, St Andrews KY16 9SS, UK.; 2 Max Planck Institute for Chemical Physics of Solids, Nöthnitzer Straße 40, 01187 Dresden, Germany.; 3 Max Planck Institute for the Physics of Complex Systems, Nöthnitzer Straße 38, 01187 Dresden, Germany.; 4 CPHT, CNRS, Ecole Polytechnique, Institut Polytechnique de Paris, F-91128 Palaiseau, France.; 5 Institut de Physique, Collège de France, 11 place Marcelin Berthelot, 75005 Paris, France.; 6 Diamond Light Source, Harwell Campus, Didcot, OX11 0DE, UK.; 7 Center for Computational Quantum Physics, Flatiron Institute, New York, NY 10010, USA.; 8 DQMP, Université de Genève, 24 quai Ernest Ansermet, CH-1211 Genève, Switzerland.

## Abstract

A nearly free electron metal and a Mott insulating state can be thought of as opposite ends of the spectrum of possibilities for the motion of electrons in a solid. Understanding their interaction lies at the heart of the correlated electron problem. In the magnetic oxide metal PdCrO_2_, nearly free and Mott-localized electrons exist in alternating layers, forming natural heterostructures. Using angle-resolved photoemission spectroscopy, quantitatively supported by a strong coupling analysis, we show that the coupling between these layers leads to an “intertwined” excitation that is a convolution of the charge spectrum of the metallic layer and the spin susceptibility of the Mott layer. Our findings establish PdCrO_2_ as a model system in which to probe Kondo lattice physics and also open new routes to use the a priori nonmagnetic probe of photoemission to gain insights into the spin susceptibility of correlated electron materials.

## Teaser

An intrinsically nonmagnetic spectroscopy is shown to have strong magnetic sensitivity in Kondo-coupled PdCrO_2_.

## INTRODUCTION

**Fig. 1:**
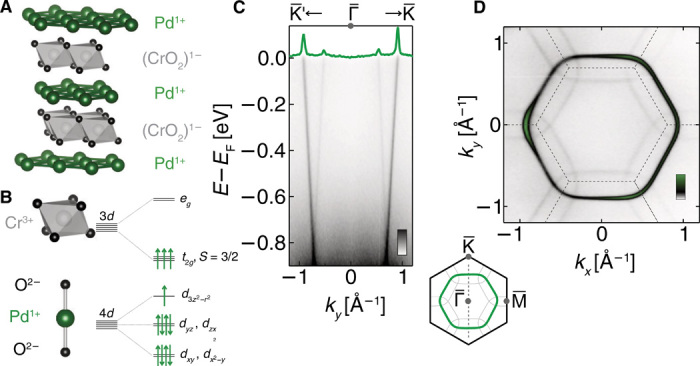
Low-energy electronic structure of PdCrO_2_. (**A**) Layered crystal structure of PdCrO_2_. (**B**) Pd layers are metallic, while the CrO_2_ layers are Mott insulating and antiferromagnetically ordered below *T*_N_ = 37.5 K. (**C**) Dispersion measured by ARPES (*h*ν = 110 eV, *T* = 6 K) along the $\bar{\Gamma }-\bar{K}$ direction (dashed line on the schematic of the crystallographic Brillouin zone) showing steep Pd-derived metallic bands, as well as replicas of these bands, apparently backfolded across the magnetic Brillouin zone boundary [dashed lines in (D)]. Notably, the observed reconstructed spectral weight is approximately energy independent over nearly 1 eV, remaining clearly visible at the Fermi level, as evident in the momentum distribution curve [green line in (C), *E*_F_ ± 5 meV], and the measured Fermi surface (**D**) (*hν* = 120 eV, *T* = 6 K, integrated over *E*_F_ ± 25 meV).

PdCrO_2_ is a member of the broad class of layered triangular lattice materials whose layer stacking sequence (see Fig. 1A) is that of the delafossite structural family ABO_2_ (*1*). In a simple ionic picture of the delafossites, triangular coordinated layers of A^+^ ions are stacked between oxygen octahedra with B^3+^ ions in the center, in which the B ions also have triangular coordination (*2*, *3*). Most delafossites are insulating or semiconducting. PdCoO_2_ and PtCoO_2_, however, are extremely high conductivity metals featuring broad conduction bands whose character is dominantly that of the A site cation Pd or Pt (*4*–*9*), with the B-site Co^3+^ cation in the band insulating and nonmagnetic 3*d* ^6^ configuration.

In contrast, in the Cr-based analog PdCrO_2_, the Cr^3+^ cations are formally in the 3*d*^3^ configuration (Fig. 1B). It was therefore not regarded as unexpected when PdCrO_2_ was observed to be magnetic, obeying a Curie-Weiss law at high temperatures, followed by a transition to 120° antiferromagnetism at a Néel temperature *T*_N_ of 37.5 K, carrying a localized spin of *S* = 3/2 (*10*–*14*). From angle-resolved photoemission (ARPES), Sobota *et al.* (*15*) found an extremely similar Fermi surface to PdCoO_2_, indicating that the low-energy electronic structure is still dominated by the Pd-derived states. A reconstruction of the Fermi surface due to the magnetic order was reported by de Haas–van Alphen measurements (*16*, *17*). Careful analysis of magnetic breakdown across the gaps opened at the antiferromagnetic (AF) Brillouin zone boundary showed that these gaps are small, on the order of 40 meV (*16*). Noh *et al.* (*18*) subsequently reported that the bands are apparently backfolded across the AF zone boundary in ARPES measurements.

## RESULTS

Our own measurements show similar spectroscopic signatures to those found by Noh *et al.* (*18*) and are evident in our own measurements shown in Fig. 1 (C and D), where weak but clear spectral weight can be observed as replicas of the “main band” [central hexagonal Pd-derived Fermi surface (*15*)] shifted in momentum by the AF ordering vector. The observation of localized 3/2 spins on the Cr sites strongly suggests that, in addition to being magnetic, the CrO_2_ layer is Mott insulating (*9*, *17*, *19*). This hypothesis has recently been supported by combined density functional theory (DFT) and dynamical mean-field theory (DMFT) calculations from our own group (text S2) and independent work by Lechermann (*20*), which concluded that in the paramagnetic state above *T*_N_, the conduction in PdCrO_2_ comes from a single band of dominantly Pd character. The Cr-derived states, predicted by standard DFT to produce additional Fermi surfaces (*20*, *21*), instead form a lower Hubbard band giving substantial incoherent spectral weight 1 to 2 eV below the Fermi level.

**Fig. 2:**
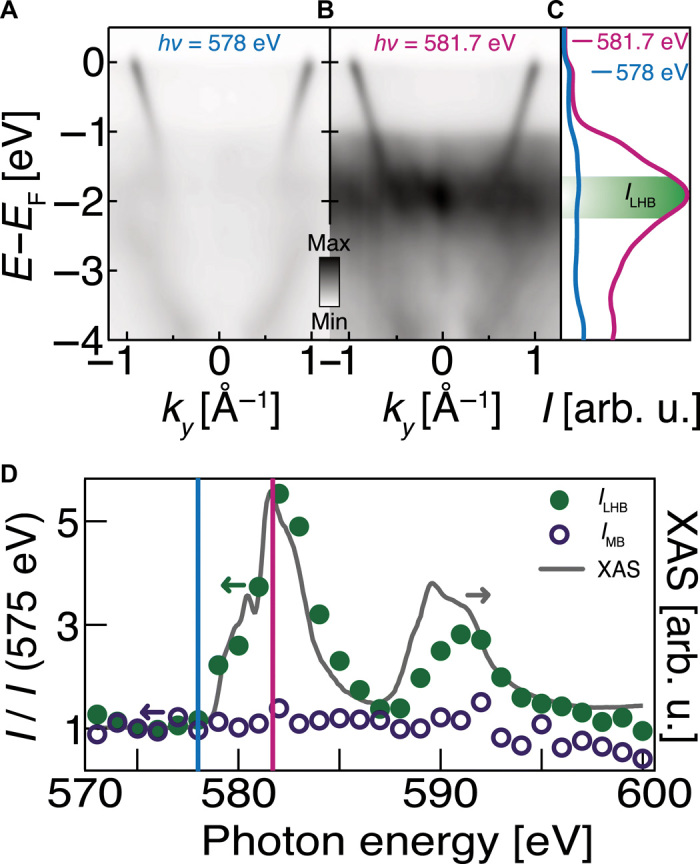
Mott insulating CrO_2_ layers. Soft x-ray ARPES (*T* = 13 K) from PdCrO_2_ at photon energies of (**A**) 578 eV and (**B**) 581.7 eV, respectively, tuned off- and on-resonance with the Cr L_3_-edge. The on-resonant spectrum reveals considerable broad spectral weight centered at approximately 2 eV below the Fermi level. The measured intensity of this feature, *I*_LHB_, extracted from energy distribution curves (**C**) (integrated over 0 ± 0.5 Å^−1^) as a function of probing photon energy is in excellent agreement with the measured x-ray absorption spectrum (XAS) across the Cr L_2,3_-edge (**D**). In contrast, the intensity of the Pd-derived main band (*I*_MB_, extracted from fits to momentum distribution curves at the Fermi level) remains approximately constant across the resonance. The data provide strong evidence that the diffuse weight visible in the ARPES measurements is dominantly of Cr character, while comparison with the DFT + DMFT calculations [see text S2 and (*20*)] identifies it as the lower Hubbard band of a Mott insulating state.

To experimentally verify this picture, we have used soft x-ray ARPES to investigate the atomically resolved electronic structure, tuning the probing photon energy into resonance with the Cr L_2,3_ absorption edge (*22*). Comparing on- to off-resonant spectra (Fig. 2, A to C) reveals a marked enhancement of spectral weight of a very weakly dispersing and broad feature centered at approximately 2 eV below *E*_F_. The integrated intensity of this feature (*I*_LHB_) tracks the Cr L_2,3_-edge x-ray absorption spectrum (Fig. 2D), thus establishing its Cr-derived origin. Comparison with the findings from the DFT + DMFT calculations provides strong evidence that this is the spectroscopic signal of a lower Hubbard band. In addition, and consistent with Fig. 1C, we observe a steeply dispersing metallic band. This band shows negligible change in spectral weight across the Cr L_2,3_-edge resonances (*I*_MB_ in Fig. 2D), confirming that it originates from the Pd layers. PdCrO_2_ can therefore be considered as an atomic layer-by-layer superlattice of a nearly free electron metal alternating with a Mott insulator.

**Fig. 3:**
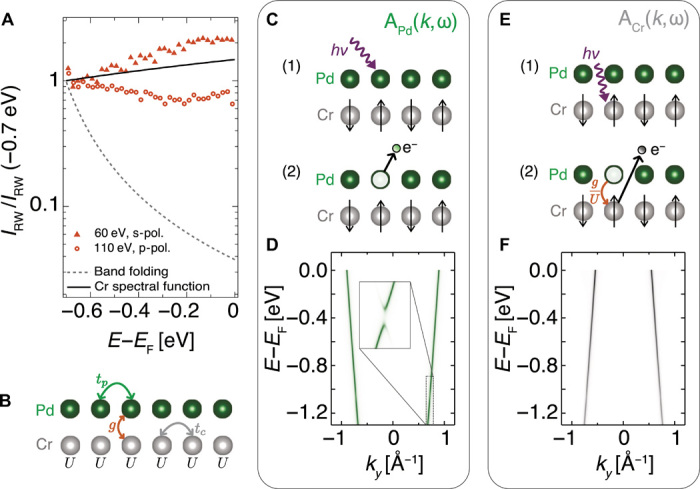
Intertwined spin and charge response. (**A**) Reconstructed weight (*I*_RW_) as obtained from fits to the dispersion shown in Fig. 1C (circles). Because of photoemission matrix elements, small quantitative variations are found when measuring using different photon energies and light polarizations. We also show here the data measured using 60 eV of s-polarized light (triangles) to illustrate the range of observed spectral weight variations; additional measurements are shown in fig. S5. In all cases, *I*_RW_ varies only weakly with binding energy. This is in sharp contrast to the simple band folding model (dashed line; see text S3.2) but in agreement with the Cr spectral function predicted by our theory (solid line; see the main text). The intensities are shown normalized to the intensity at a binding energy of −0.7 eV to aid judging the relative binding energy–dependent variations in the data and different models; equivalent conclusions are drawn if normalizing directly by the main band intensity (fig. S7). (**B**) The starting point of the theory is a Hamiltonian that includes hopping within (*t_p_*, *t_c_*) and between (*g*) the layers, as well as the on-site Coulomb repulsion on the Cr sites (*U*). (**C**) Schematic illustration of photoemission of Pd electrons. (**D**) The corresponding spectral function is equivalent to that predicted by the band folding model. (**E**) Photoemission of a Cr electron can proceed via a virtual process involving tunneling of the Cr hole to the Pd layer. (**F**) This results in a spectral function that is a convolution of the Pd spectrum and the spin correlation function of the Mott layer (Eq. 3), thus appearing as a copy of the Pd spectral function shifted by the wave vector of the AF order, in agreement with the experiment (Fig. 1C).

Given the AF order of the latter, the observation of replicas of the metallic main band shown in Fig. 1 (C and D) might, at first sight, seem unremarkable. In general, when electrons feel an additional periodic potential, for example, due to a density wave or magnetic order, the band structure is reconstructed. Replica bands appear, shifted from the original ones by the wave vectors of the new potential, with hybridization gaps opening at the new Brillouin zone boundaries. This standard picture, however, cannot explain the experimental observations of PdCrO_2_. The spectral weight of the replicas observable by ARPES should fall off rapidly away from the new zone boundaries (*23*), with a form equivalent to the well-known coherence factors of Bogoliubov quasiparticles in a superconductor. Experimentally, however, the replicas are clearly observed all the way from the magnetic zone boundary to the Fermi level (Fig. 1, C and D), an energy range more than an order of magnitude larger than the hybridization gap scale of ~40 meV (*16*). Over the same energy range, the simple “band folding” model predicts a 100-fold decrease in spectral weight (dashed line in Fig. 3A), which would render the backfolded bands invisible to ARPES. In contrast, the measured intensity of the reconstructed weight (*I*_RW_) changes by less than a factor of 2 (symbols in Fig. 3A). Additional measurements performed using different light polarization and photon energies show similarly weak binding energy dependence of the reconstructed weight (fig. S5); the striking discrepancy with the band folding model cannot, therefore, be explained by photoemission matrix element variations. A further possibility worthy of investigation is final-state Umklapp scattering, in which an outgoing photoelectron is diffracted from the potential of a superperiodic structure during its travel to the surface. This can, in principle, yield backfolded bands whose spectral weight has only a weak binding energy dependence (*24*–*27*). In the delafossites, however, this possibility can be ruled out by making a direct comparison with the isostructural, but nonmagnetic, PdCoO_2_. Under identical measurement conditions to those of PdCrO_2_, we observe no such signal (fig. S3), and so, it is clear that the replica features of Fig. 1 (C and D) require a different explanation.

We have discovered that the answer to the above puzzle lies in Mott insulator–free electron coupling. Rather than treating the CrO_2_ layer as a passive source of a periodic potential, it is necessary to take into account its dynamical degrees of freedom. To illustrate this, we start with a minimal model (shown schematically in Fig. 3B) combining hopping within and between the Pd and CrO_2_ layers with the Coulomb repulsion in the Mott layer

$$\begin{array}{c} H=-\sum_{\mathrm{ij}\sigma }^{n.n.}(t_{p}p_{i\sigma }^{†}p_{j\sigma }+t_{c}c_{i\sigma }^{†}c_{j\sigma })+U\sum_{i}(n_{i↑}^{c}-\frac{1}{2})(n_{i↓}^{c}-\frac{1}{2})+\\ \sum_{\mathrm{ij}\sigma }^{n.n.}g_{\mathrm{ij}}(p_{i\sigma }^{†}c_{j\sigma }+H.c.) \end{array}$$ (1)

where *t_p_* (*t_c_*) denotes the hopping integrals between the Pd (Cr) sites, *g* is the interlayer hopping, and *U* is the Coulomb repulsion. We neglect Coulomb repulsion between the Pd electrons, justified by the fast band velocities of the Pd-derived states observed experimentally (see fig. S3C). Here, we omit the orbital indices and assume *S* = 1/2 on the Cr sites for conceptual simplicity. We present the full multiorbital model with *S* = 3/2, the results of which are shown in Fig. 3 (D and F) and text S3.

The large size of *U* compared to the other coupling constants enables a standard strong coupling analysis, implemented via a Schrieffer-Wolff transformation (full procedure is described in text S3) to derive a low-energy Kondo lattice Hamiltonian

$$H_{\text{eff}}=-t_{p}\sum_{\mathrm{ij}\sigma }^{n.n.}p_{i\sigma }^{†}p_{j\sigma }+\frac{4t_{c}^{2}}{U}\sum_{\langle \mathrm{ij}\rangle }^{n.n.}S_{i}\cdot S_{j}+\frac{4}{U}\sum_{\mathrm{ijk}\sigma \sigma \prime }^{n.n.}g_{\mathrm{ij}}g_{\mathrm{kj}}p_{i\sigma }^{†}(S_{j}\cdot \sigma_{\sigma \sigma \prime })p_{k\sigma \prime }$$ (2)

in which the second term captures the effective spin-spin exchange in the Mott layer and the last term describes a Kondo coupling between the localized Cr spin and Pd electrons on the neighboring sites.

The Hamiltonian (Eq. 2) is of a standard form but applied here to an unusual situation in which the Kondo coupling is an interlayer effect. The key insights it provides come from using it to calculate the spectral functions for the photoemission process. The coupling allows the Pd electrons to feel the periodic potential due to the AF order of the Cr spins but does not otherwise affect their basic itinerant nature. The resulting Pd one-electron removal spectral function (Fig. 3, C and D) thus, as expected, looks like the simple band folding model introduced above: It largely follows the unperturbed Pd dispersion, with small gaps opening at the magnetic zone boundary as seen by quantum oscillations (*16*), and has a weak, strongly energy-dependent weight in the reconstructed band.

In contrast, the removal of electrons from Cr orbitals is markedly altered by the coupling to the Pd layer. It would be impossible to remove an electron from an isolated Mott layer at energies smaller than *U*. However, for finite interlayer coupling *g*, a hole created in the Mott layer can rapidly move to the itinerant layer where it can propagate; formally, the Schrieffer-Wolff transformation leads to an effective real space Cr removal operator of the form ${\left(c_{j\sigma }\right)}_{\text{eff}}=\frac{2}{U}\;\sum_{k}^{n.n.\;\text{of}\;j}g_{\mathrm{kj}}(S_{j}\cdot \sigma_{\sigma \sigma \prime })p_{k\sigma \prime }$. We note two important features of the transformed operator. First, the process is perturbatively small in *g*/*U*. Second, it provides a connection between the itinerant Pd electrons and Mott spins. This results in the spectral function for the removal of electrons from the Mott layer becoming a convolution of the itinerant electron spectrum with the spin correlation function of the Mott layer

$$\begin{array}{c} A_{\text{Cr}}(k,\omega <0)=-\int_{-\infty }^{0}\frac{d\omega \prime }{2\pi }\int \frac{d^{3}q}{{\left(2\pi \right)}^{3}}\frac{32{|g_{k+q}|}^{2}}{U^{2}}\\ A_{\text{Pd}}(k+q,\omega \prime )\langle S_{q}\cdot S_{-q}(\omega -\omega \prime )\rangle \end{array}$$ (3)

In this way, the spin response of the Mott layer and the charge response of the itinerant layer become intertwined. In the case of AF ordered PdCrO_2_, the mean-field spin correlation function is a δ function at zero energy and the AF wave vector. The resulting prediction (Fig. 3, E and F) is that Cr spectral weight now exists at energies much lower than *U* and that it follows the dispersion of the nearly free electron Pd band but translated by the wave vector of the AF order.

This calculation of intertwined spin-charge response yields at least two testable predictions for the observable spectral properties that are qualitatively different from those of a standard band folding model. First, as discussed above, standard band folding predicts a spectral weight of the replica band that dies off extremely quickly with energy away from the magnetic Brillouin zone boundary. In contrast, the intertwined spin-charge model has no inherent energy dependence of the reconstructed weight; momentum dependence of the interlayer coupling constant *g* can still give small system-specific variations (text S3.2 and fig. S6), but in general, the energy dependence of the reconstructed weight will be weak. As shown in Fig. 3A, observation is in close agreement with the latter prediction. The second and even more notable prediction is that the backfolded spectral weight, despite appearing as a sharp band-like feature, is actually a property of the Cr removal spectral function rather than the Pd one. This is, at first sight, highly unexpected, because “Mott electrons” are associated with the broad incoherent spectral weight displayed and described in Fig. 2.

**Fig. 4:**
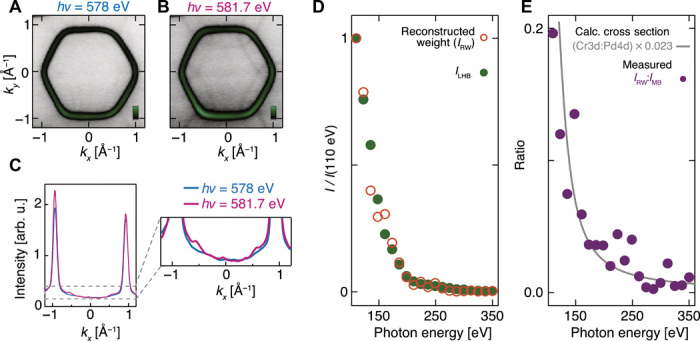
Cr origin of the reconstructed weight. The Fermi surface measured (*T* = 13 K, integrated over *E*_F_ ± 200 meV) off-resonance (**A**) (*h*ν = 578 eV) and on-resonance (**B**) (*hν* = 581.7 eV) with the Cr L_3_-edge. The reconstructed weight is markedly enhanced in the resonant condition, as evident in a comparison of momentum distribution curves at the Fermi level recorded on- and off-resonance (**C**). (**D**) The photon energy dependence of the reconstructed weight (*I*_RW_) at lower photon energies closely tracks that of the Cr-derived lower Hubbard band (*I*_LHB_). (**E**) The ratio of *I*_RW_ to the weight of the main band (*I*_MB_) is strongly photon energy dependent. It follows the functional form expected for the Cr 3*d*:Pd 4*d* ionic cross-section ratio (*30*), scaled by a factor of ~0.023, the origin of which is the spectral weight suppression factor of (*g*/*U*)^2^ predicted by the intertwined spin-charge model (Eq. 3).

The key diagnostic for the validity of the intertwined spin-charge model is, therefore, to establish the underlying atomic origin of the reconstructed spectral weight. To do this, we again use soft x-ray ARPES to show that the reconstructed weight (*I*_RW_) is markedly enhanced when the photon energy is tuned to the Cr L_3_-edge resonance (Fig. 4, A to C). Moreover, quantitative analysis of measurements performed at lower photon energies shows (i) that the photon energy dependence of the reconstructed weight closely traces that of the Cr-derived lower Hubbard band (Fig. 4D) and (ii) that its ratio to the Pd-derived main band intensity tracks the Cr 3*d*:Pd 4*d* ionic cross- section ratio (Fig. 4E). These observations all point to a dominant Cr character of the backfolded spectral weight.

Equation 3 for the Cr spectral function suggests that the weight of the reconstructed feature should be suppressed by approximately 32*g*^2^/*U*^2^ as compared to the weight of the main band in the Pd spectral function. While ARPES matrix elements prevent us from making a direct quantitative measurement of the intrinsic relative weights, comparison with the ionic cross-section ratio shown in Fig. 4D indicates that their ratio is on the order of 1%. With *U* = 4 eV, the interlayer coupling *g* is thus estimated to be on the order of 100 meV. This is similar to values derived from a DFT analysis of interlayer hopping (table S1), providing a further consistency check on our analysis.

In principle, a standard one-electron Pd-Cr hybridization could lead to a Cr character of the replica weight. This would, however, be fundamentally incompatible with the lack of binding energy dependence of its measured spectral weight. Obtaining measurable Cr weight up to the Fermi level would require hybridization gaps on the order of ~1 eV to open where the bands cross (*18*), which is completely inconsistent with the measured electronic structure both from ARPES (Fig. 1, C and D) and quantum oscillations (*16*). On the other hand, while a final-state Umklapp process, as introduced above, could explain the binding energy–independent reconstructed weight, it could not explain its Cr-derived character: If it were due to a final-state Umklapp of the Pd-derived main band of either structural or exchange scattering origin, then it would exhibit Pd character and not the Cr character that we observe experimentally. The combination of these two key experimental observations (weak binding energy dependence and Cr character of the reconstructed weight) thus provides compelling evidence that the spectroscopic information obtained from ARPES measurements on PdCrO_2_ is determined by a Kondo coupling of nearly free electrons in metallic layers, with localized electrons in a Mott insulating state in adjacent layers.

## DISCUSSION

The realization that the spectroscopic information in PdCrO_2_ is determined by a Kondo coupling between nearly free electrons in metallic layers with localized electrons in a Mott insulating state in adjacent layers is, we believe, exciting for a number of reasons. It establishes PdCrO_2_ as a benchmark system for studies of the triangular lattice Hubbard model and Mott insulator–free electron coupling. The transport properties of PdCrO_2_ (*10*–*14*, *17*) are of considerable interest in their own right and invite further theoretical work. In Eqs. 1 and 2 above, and in more detail in supplementary text S3, we present model Hamiltonians (including experimentally constrained bare parameters) as a foundation for such calculations. However, we believe that our findings are also of considerable relevance across broader fields of research.

The first regards the interplay of localized and itinerant electrons in solids in general, which lies at the heart of the physics of Kondo systems. PdCrO_2_ lies in the AF metal region of the famous Doniach phase diagram (*28*) because the localized spin is underscreened. Although this means that the formation of heavy fermions is not expected in the current experimental situation, the insights that we have uncovered open new ways to study systems from across the phase diagram, even in the Kondo limit. This would, in principle, include quasi–three-dimensional materials in which the layer-dependent coupling in PdCrO_2_ is replaced by, for example, a coupling between localized *f*-electrons and itinerant conduction electrons. The limitation, in practice, will be one of resolution; if the bandwidths become too small and the *k*_z_ dispersion becomes too strong, then the spin-related signatures will be harder to extract, but such information should exist in the experimental signal. We also stress that the analysis presented above does not rely on the existence of a static magnetic order, and could equally well apply to a disordered system with a peaked susceptibility, of the type discussed in the context of cuprate superconductors (*29*). In that case, the width of the feature observed in photoemission would be related to the AF correlation length and time.

In such a dynamical case, because of the way in which magnetic and charge excitations associated with different subsystems are intertwined during the photoemission of electrons from the Mott subsystem, we can obtain information on both in a single measurement. There may be a regime of coupling in which separated magnon and charge velocities could become observable by ARPES. Our work, therefore, has a further, conceptually attractive component in providing a complementary perspective to the physics of spin-charge separation. There, an electron in a solid fractionalizes into independently mobile excitations carrying its magnetic and charge quantum numbers. Another interesting avenue for future theoretical research is to investigate how the existence of a Mott state, which is itself fractionalized, would be manifested in this type of experiment.

Our findings thus point to the broad potential of combining itinerant and Mott insulating systems in heterostructure geometries, where our analysis indicates that finite coupling between the layers leads to new physics not present in either of the spatially separated subsystems alone. Consistent with this, a recent DMFT study (*20*) found that doping of the Mott layer in PdCrO_2_ results in an effective Pd layer doping. Although the underlying process is not the same as in the photoemission problem studied here, both effects reflect the fact that in a coupled Mott/itinerant system, it is the itinerant layer that will support charge excitations.

The insights gained here therefore have relevance to the study of materials well beyond PdCrO_2_. For example, while the development of spin-resolved detectors has opened opportunities to study spin-polarized itinerant bands, ARPES is typically not expected to be sensitive to finite ***q*** local moment magnetism: Our findings show that this need not be true. Our work thus opens an entirely new route to investigating both static and dynamical spin susceptibilities in correlated solids, including systems that are inaccessible to more traditional magnetic probes such as neutron scattering. Combining ARPES studies with targeted materials design of coupled Mott-metal heterostructures could therefore provide unique information on magnetic ordering and fluctuations in previously unexplored regimes in, for example, transition-metal oxide heterostructures, two-dimensional van der Waals magnetic insulators, and candidate quantum spin liquids. Our work thus motivates the study and creation of interfaces between metals and Mott insulators, not only to provide routes to probe the spin correlations of the correlated subsystem but also as a platform for stabilizing and observing new physics.

## MATERIALS AND METHODS

Single-crystal samples of PdCrO_2_ were grown by a NaCl-flux method in sealed quartz tubes as described in (*11*). They were cleaved in situ at the measurement temperature of 6 to 13 K. High-resolution ARPES measurements (Figs. 1, C and D, and 3A and fig. S1) were performed at the I05 beamline of Diamond Light Source, UK, using a Scienta R4000 hemispherical electron analyzer. The spectra shown in Fig. 1, C, D were measured using p-polarized light, while fits to data taken using both s- and p-polarized light are included in Fig. 3A and fig. S5. The soft x-ray measurements (Figs. 2 and 4) were performed with p-polarized light at the I09 beamline of Diamond Light Source, UK; the ARPES measurements were performed using a Specs Phoibos 225 hemispherical electron analyzer, while the x-ray absorption was recorded in the total electron yield mode and was normalized by the photon flux. Further details of the theoretical methods are described in Supplementary Text.

## Supplementary Material

http://advances.sciencemag.org/cgi/content/full/6/6/eaaz0611/DC1

Download PDF

Probing spin correlations using angle-resolved photoemision in a coupled metallic/Mott insulator system

## SUPPLEMENTARY MATERIALS

Supplementary material for this article is available at http://advances.sciencemag.org/cgi/content/full/6/6/eaaz0611/DC1 Text S1. Density functional theory. Text S2. DFT + DMFT calculations: Spectral function. Text S3. Strong coupling theory. Fig. S1. Comparison of the DFT band structure and the Wannier function–based 10-band tight-binding model near the Fermi level. Fig. S2. DFT + DMFT electronic structure calculations. Fig. S3. Comparison of electronic structure in PdCoO_2_ and PdCrO_2_. Fig. S4. Schematic picture of the Cr 3*d* orbitals. Fig. S5. Exchange pathways in PdCrO_2_. Fig. S6. Binding energy–dependent spectral weight. Fig. S7. The ratio of the reconstructed weight and the main band weight. Table S1. Table of hopping parameters. Table S2. Table of spin coupling constants derived from the strong coupling expansion. Table S3. Cr-Cr superexchange interactions calculated by the DFT + DMFT method. References (*31*–*42*)
