# Supplementary material for: Probing spin correlations using angle-resolved photoemission in a coupled metallic/Mott insulator system
Source: Sci Adv. 2020 Feb 7;6(6):eaaz0611. doi: 10.1126/sciadv.aaz0611 (PMC7032925; doi:10.1126/sciadv.aaz0611)
Supplement: http://advances.sciencemag.org/cgi/content/full/6/6/eaaz0611/DC1 [file supp_6_6_eaaz0611__index.html]

Science Advances | Science AdvancesAAASSearchScience AdvancesMenu

## Supplementary Materials

**This PDF file includes:**

- Text S1. Density functional theory.
- Text S2. DFT + DMFT calculations: Spectral function.
- Text S3. Strong coupling theory.
- Fig. S1. Comparison of the DFT band structure and the Wannier function–based 10-band tight-binding model near the Fermi level.
- Fig. S2. DFT + DMFT electronic structure calculations.
- Fig. S3. Comparison of electronic structure in PdCoO2 and PdCrO2.
- Fig. S4. Schematic picture of the Cr 3*d* orbitals.
- Fig. S5. Exchange pathways in PdCrO2.
- Fig. S6. Binding energy–dependent spectral weight.
- Fig. S7. The ratio of the reconstructed weight and the main band weight.
- Table S1. Table of hopping parameters.
- Table S2. Table of spin coupling constants derived from the strong coupling expansion.
- Table S3. Cr-Cr superexchange interactions calculated by the DFT + DMFT method.
- References (*31*–*42*)

Download PDF

**Files in this Data Supplement:**

- Adobe PDF - aaz0611\_SM.pdf
