# Supplementary material for: Probing spin correlations using angle-resolved photoemission in a coupled metallic/Mott insulator system
Source: Sci Adv. 2020 Feb 7;6(6):eaaz0611. doi: 10.1126/sciadv.aaz0611 (PMC7032925; doi:10.1126/sciadv.aaz0611)
Supplement: Download PDF [file aaz0611_SM.pdf]

Science Advances
AAAS

advances.sciencemag.org/cgi/content/full/6/6/eaaz0611/DC1

Supplementary Materials for

Probing spin correlations using angle-resolved photoemission in a coupled metallic/Mott insulator system

V. Sunko, F. Mazzola, S. Kitamura, S. Khim, P. Kushwaha, O. J. Clark, M. D. Watson, I. Marković, D. Biswas, L. Pourovskii, T. K. Kim, T.-L. Lee, P. K. Thakur, H. Rosner, A. Georges, R. Moessner, T. Oka*, A. P. Mackenzie*, P. D. C. King*

*Corresponding author. Email: oka@pks.mpg.de (T.O.); andy.mackenzie@cpfs.mpg.de (A.P.M.); philip.king@st-andrews.ac.uk (P.D.C.K.)

Published 7 February 2020, *Sci. Adv.* **6**, eaaz0611 (2020)
DOI: 10.1126/sciadv.aaz0611

The PDF file includes:

Text S1. Density functional theory.
Text S2. DFT + DMFT calculations: Spectral function.
Text S3. Strong coupling theory.
Fig. S1. Comparison of the DFT band structure and the Wannier function–based 10-band tight-binding model near the Fermi level.
Fig. S2. DFT + DMFT electronic structure calculations.
Fig. S3. Comparison of electronic structure in $PdCoO_2$ and $PdCrO_2$.
Fig. S4. Schematic picture of the Cr 3*d* orbitals.
Fig. S5. Exchange pathways in $PdCrO_2$.
Fig. S6. Binding energy–dependent spectral weight.
Fig. S7. The ratio of the reconstructed weight and the main band weight.
Table S1. Table of hopping parameters.
Table S2. Table of spin coupling constants derived from the strong coupling expansion.
Table S3. Cr-Cr superexchange interactions calculated by the DFT + DMFT method.
References (*31*–*42*)

Supplementary Text

Text S1. Density functional theory.

Scalar-relativistic density functional theory (DFT) electronic structure calculations were performed using the full-potential FPLO code (*31–33*), version fplo18.00-52. For the exchange-correlation potential, within the local density approximation, the parameterization of Perdew-Wang (*34*) was chosen. To obtain precise band structure, the calculations were carried out on a0020well converged mesh of 8000 *k*-points (20x20x20 mesh, 781 points in the irreducible wedge of the Brillouin zone). For all calculations, the experimental crystal structure was used *(13)*.

From the converged calculation, a 10-band tight-binding model (TBM) based on Wannier functions was constructed in an energy window between -3 eV and 2 eV. For the TBM, all Cr 3*d* and Pd 4*d* orbitals were taken into account. This results in very good agreement with the DFT bands in the relevant energy window near the Fermi level (see fig. S1). The extracted tight binding integrals and on-site energies (see Table S1) were used as input parameters for the strong coupling theory (see Text S3).

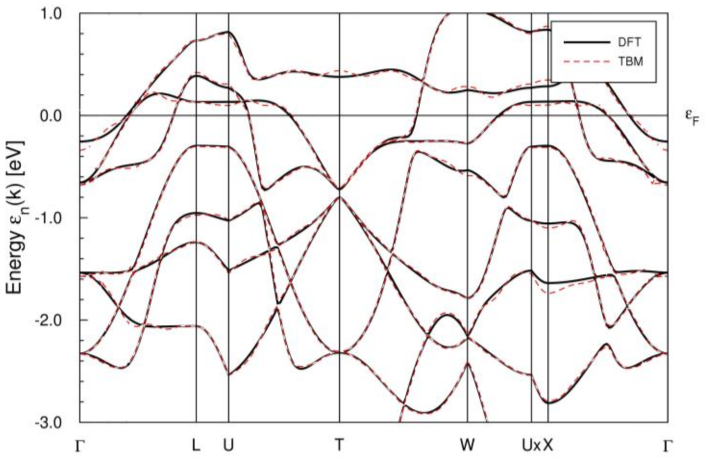

Fig. S1. Comparison of the DFT band structure (full black lines) and the Wannier function–based 10-band tight-binding model (red dashed lines) near the Fermi level. Two ghost bands that have only weight for the unoccupied Cr 3*d* e_g manifold have been removed for clarity.

Text S2. DFT + DMFT calculations: Spectral function

DFT + dynamical mean-field theory (DMFT) calculations for $PdCrO_2$ were carried for its experimental lattice structure (a=2.92 Å, c=18.09 Å) with a charge self-consistent framework (*35*, *36*) combining the linearized augmented planewave band-structure code "Wien2k" (*37*) and the DMFT implementation provided by the library "TRIQS" (*38*, *39*). Projective Wannier orbitals representing Cr 3d and Pd 4d states were constructed from Kohn-Sham bands in the energy range of [-5:11] eV. The rotationally-invariant on-site Coulomb repulsion vertex between Cr 3d orbitals was specified by the Slater parameter F^0 = 4.5 eV and Hund's rule coupling J_H = 0.75 eV. We solved the DMFT quantum impurity problem employing the quasi-atomic Hubbard-I approximation (*40*). The double-counting correction was evaluated in the fully-localized limit

using the atomic occupancy 3 of the Cr $3d$ shell. Self-consistent DFT+DMFT calculations were converged to 0.05 mRy in the total energy.

The resulting total $\boldsymbol{k}$-resolved DFT+DMFT spectral function $A(\boldsymbol{k}, \omega)$ is displayed in fig. S2. Within the Hubbard-I approximation the DMFT self-energy has no imaginary part, hence, there is also no lifetime broadening in our resulting $A(\boldsymbol{k}, \omega)$. Apart from this, however, our calculated spectral function is in excellent agreement with that obtained in Ref. *(20)* using a numerically exact quantum Monte Carlo method (one may notice a rather weak lifetime broadening for the occupied part of $A(\boldsymbol{k}, \omega)$ shown in Fig. 3 of Ref. *(20)*).

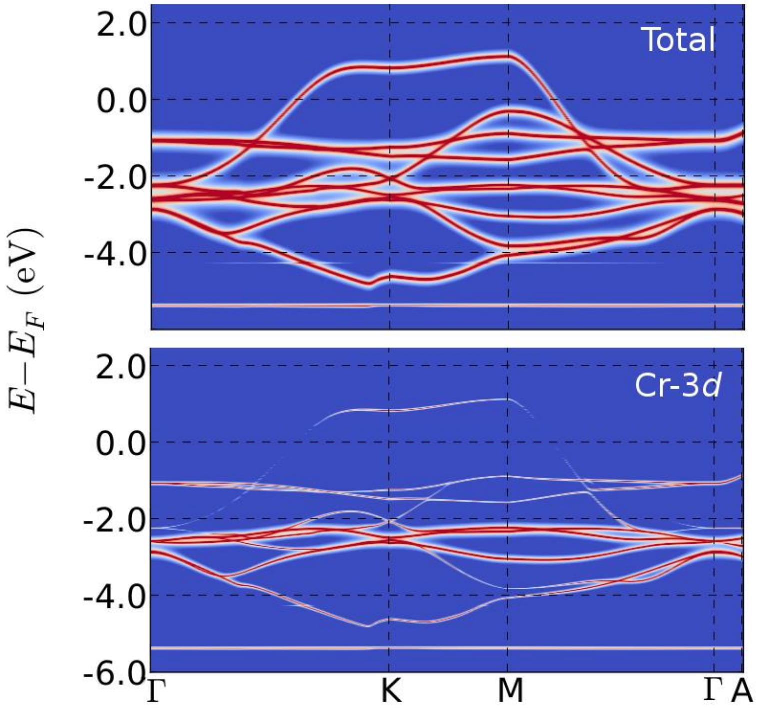

Fig. S2. DFT + DMFT electronic structure calculations. Total (upper panel) and projected Cr 3*d* (low panel) *k*-resolved DFT+DMFT spectral function $A(\boldsymbol{k}, \omega)$ of $PdCrO_2$.

The largest contribution of the Cr 3*d* character to the DFT+DMFT $A(\boldsymbol{k}, \omega)$ is seen in the range from -2.5 to -2 eV, in good agreement with the location of diffuse spectral weight in the experimental on-resonance ARPES (Fig. 2B of the main text). There is almost no Cr contribution to the dispersive bands crossing E_F, confirming that they are of dominant Pd 4*d* character. This finding is supported by our ARPES measurements: as well as a lack of spectral-weight enchancement of this band across the Cr $L_{2,3}$ – edge resonances (I_{MB} in Fig. 2D of the main text), our high-resolution measurements indicate that its Fermi velocity is very similar in $PdCrO_2$ and the non-magnetic sister compound $PdCoO_2$, and very fast in both (fig. S3). This rules out any significant correlation-driven mass enhancement of these states.

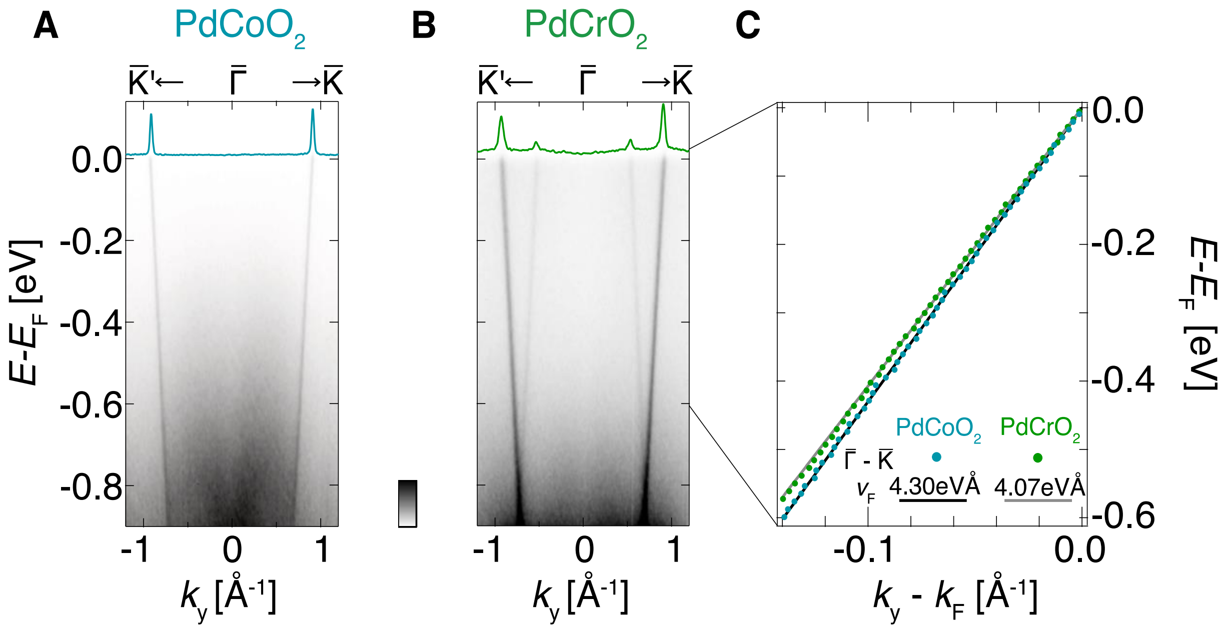

Fig. S3. Comparison of electronic structure in $PdCoO_2$ and $PdCrO_2$. Dispersion measured by ARPES (hv = 110 eV, T = 6 K) along the $\overline{\Gamma} - \overline{K}$ direction of (**A**) $PdCoO_2$ and (**B**) $PdCrO_2$. (**C**) Fits to MDCs in the vicinity of the Fermi level for both compounds, showing fast Fermi velocities (linear fits). In $PdCrO_2$ the weak interplane coupling renormalises the Fermi velocity by at most 6% as compared to $PdCoO_2$, although a slight lattice expansion for $PdCrO_2$ vs. $PdCoO_2$ means that the actual degree of correlation-driven mass enhancement is likely even smaller than this value. This justifies the treatment of the Pd electrons as uncorrelated in the intertwined spin-charge model discussed in the main text.

Text S3. Strong coupling theory

The demonstration of a Mott insulating state of the CrO_2 layers justifies the strong-coupling theory for the spectral function of the $PdCrO_2$ utilised here. While we show a simplified version without the orbital degree of freedom in the main text, here we employ a more realistic four-orbital model

$$H = -\sum_{ij\sigma} t_{ij}^{p} p_{i\sigma}^{\dagger} p_{j\sigma} - \sum_{ijmm'\sigma} t_{ij}^{mm'} c_{im\sigma}^{\dagger} c_{jm'\sigma} + \sum_{ijm\sigma} \left(g_{ij}^{m} p_{i\sigma}^{\dagger} c_{jm\sigma} + g_{ij}^{m*} c_{jm\sigma}^{\dagger} p_{i\sigma} \right) + H_{\text{int}} \quad (1)$$

where $p_{j\sigma}$ represents the $d_{3z^2-r^2}$ orbital of Pd electrons on the jth site, while $c_{jm\sigma}$ represents the $3d$ orbitals of Cr electrons. $m = 1,2,3$ labels the three low-energy states of the crystal field splitting

$$|m=1\rangle = |d_{3z^2-r^2}\rangle, \qquad |m=2\rangle = \cos\phi\,|d_{x^2-y^2}\rangle + \sin\phi\,|d_{xz}\rangle,$$
$$|m=3\rangle = \sin\phi\,|d_{yz}\rangle - \cos\phi\,|d_{xy}\rangle \quad (2)$$

with $\tan\phi \sim 0.693$, which are schematically drawn in fig. S3. Pd and Cr respectively form triangular lattices and are stacked alternately. The hopping parameters are estimated from the 10-orbital (Pd $4d$ and Cr $3d$ electrons) model constructed from the first-principles calculations (see Text S1), and are summarized in Table S1. In reality, the metallic Pd band of the $d_{3z^2-r^2}$ orbital is substantially hybridized with d_{xy} and $d_{x^2-y^2}$ orbitals. While the multi-orbital treatment is necessary for orbitally-resolved properties (such as the Berry curvature [arXiv:1811.03105]), it is not crucial for reproducing the parabolic energy spectrum and so we neglected the detailed orbital components of Pd electrons for simplicity. The derivation of the Kondo Hamiltonian and the associated spectral formula is streightforwardly applicable to the case where the Pd electrons have the orbital indices as well.

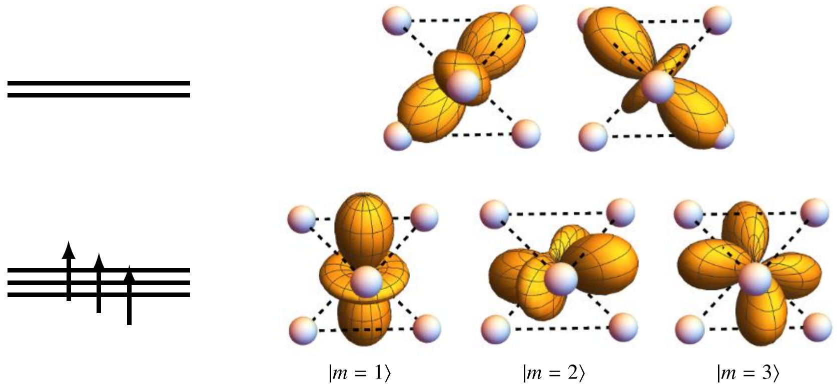

Fig. S4. Schematic picture of the Cr 3*d* orbitals. The three lower energy states are labeled as $m = 1,2,3$.

We explicitly include the onsite repulsion U and the Hund coupling J_H for the Cr electrons as

$$H_{\text{int}} = \left(\frac{1}{2}U - \frac{5}{4}J_H\right)\sum_i\left[\sum_m(n_{im} - 1)\right]^2 + \frac{1}{2}J_H\sum_{im}(n_{im} - 1)^2 - J_H\sum_i \boldsymbol{S}_i^2 \quad (3)$$

to capture the correlated nature of the $3d$ electrons. Here $n_{im} = \sum_\sigma c_{im\sigma}^\dagger c_{im\sigma}$ and

$$\boldsymbol{S}_i = \frac{1}{2}\sum_{m\sigma\sigma'} c_{im\sigma}^\dagger \boldsymbol{\sigma}_{\sigma\sigma'} c_{im\sigma'} \quad (4)$$

is the localized spin of Cr.

Table S1. Table of hopping parameters.

$\boldsymbol{R}_j - \boldsymbol{R}_i$	$\pm\sqrt{3}(0,1,0)$	$\pm\sqrt{3}(\frac{\sqrt{3}}{2},\frac{1}{2},0)$	$\pm\sqrt{3}(\frac{\sqrt{3}}{2},-\frac{1}{2},0)$	$\pm 3(1,0,0)$	$\pm 3(\frac{1}{2},\frac{\sqrt{3}}{2},0)$	$\pm 3(\frac{1}{2},-\frac{\sqrt{3}}{2},0)$
t_{ij}^{p} (meV)	568			-108		

$\boldsymbol{R}_j - \boldsymbol{R}_i$	$\pm\sqrt{3}(0,1,0)$	$\pm\sqrt{3}(\frac{\sqrt{3}}{2},\frac{1}{2},0)$	$\pm\sqrt{3}(\frac{\sqrt{3}}{2},-\frac{1}{2},0)$
$t_{ij}^{mm'}$ (meV)	$\begin{pmatrix} 141 & 0 & 152 \\ 0 & 17.6 & 0 \\ 152 & 0 & -44.9 \end{pmatrix}$	$\begin{pmatrix} 48.4 & -53.4 & -76.1 \\ -53.4 & 110 & 132 \\ -76.1 & 132 & -44.9 \end{pmatrix}$	$\begin{pmatrix} 48.4 & 53.4 & -76.1 \\ 53.4 & 110 & -132 \\ -76.1 & -132 & -44.9 \end{pmatrix}$

$\boldsymbol{R}_j - \boldsymbol{R}_i$	$\pm(-1,0,\frac{1}{2})$	$\pm(\frac{1}{2},-\frac{\sqrt{3}}{2},\frac{1}{2})$	$\pm(\frac{1}{2},\frac{\sqrt{3}}{2},\frac{1}{2})$	$\pm(2,0,\frac{1}{2})$	$\pm(-1,\sqrt{3},\frac{1}{2})$	$\pm(-1,-\sqrt{3},\frac{1}{2})$
g_{ij}^{m} (meV)	$\begin{pmatrix} 110 \\ 0 \\ -52.6 \end{pmatrix}$	$\begin{pmatrix} -55.3 \\ 95.7 \\ -52.6 \end{pmatrix}$	$\begin{pmatrix} -55.3 \\ -95.7 \\ -52.6 \end{pmatrix}$	$\begin{pmatrix} 122 \\ 0 \\ 122 \end{pmatrix}$	$\begin{pmatrix} -60.8 \\ 105 \\ 122 \end{pmatrix}$	$\begin{pmatrix} -60.8 \\ -105 \\ 122 \end{pmatrix}$

3.1 Derivation of the Kondo lattice Hamiltonian

Since the presence of the Mott transition of Cr electrons is demonstrated in the DFT+DMFT calculation (see Text S2) we perform the strong coupling expansion to investigate the low-energy structure of the Mott insulating phase. We derive an effective Hamiltonian by applying the Schrieffer-Wolff transformation

$$H_{\rm eff} = e^{i\mathcal{S}} H e^{-i\mathcal{S}} = H + [i\mathcal{S}, H] + \frac{1}{2}\big[i\mathcal{S}, [i\mathcal{S}, H]\big] + \cdots \quad (5)$$

where $\mathcal{S}$ is determined such that terms changing the number of doublon, holon, or $\boldsymbol{S}_i^2 = S_i(S_i+1)$ do not appear in the effective Hamiltonian. The Schrieffer-Wolff transformation thus `eliminates' the high energy charge (doublon-holon) excitation of the Cr atoms. The upper and lower Hubbard bands, the latter of which is visible in experiment (Fig. 2(B,C) of the main text) is thus missing in the Kondo-Heisenberg model. However, all excitations involving the spin degree of freedom are kept in the model after the transformation. We consider a perturbative expansion from the atomic limit and construct $\mathcal{S}$ order by order: We expand

$$\mathcal{S} = \mathcal{S}^{(1)} + \mathcal{S}^{(2)} + \cdots$$

and compare each order, where hopping terms are allocated to the first order and interacting terms the zeroth order. From this condition, the first-order term $\mathcal{S}^{(1)}$ must satisfy

$$i\mathcal{S}^{(1)}|GS\rangle = \frac{1}{U_{\rm eff}} \sum_{ijm\sigma} \Big[2\big(g_{ij}^{m} p_{i\sigma}^{\dagger} c_{jm\sigma} + g_{ij}^{m*} c_{jm\sigma}^{\dagger} p_{i\sigma} \big) - \sum_{m'} t_{ij}^{mm'} c_{im\sigma}^{\dagger} c_{jm'\sigma} \Big] |GS\rangle \quad (6)$$

where $|GS\rangle$ is the (macroscopically-degenerate) ground state (no doublons/holons, $S = 3/2$ on each site) of $H_{\rm int}$ and $U_{\rm eff} = U + 2J_H$. We obtain the second-order effective Hamiltonian (up to constants) from this relation as

$$H_{\rm eff} = -\sum_{ij\sigma} t_{ij}^{p} p_{i\sigma}^{\dagger} p_{j\sigma} + \frac{1}{2}\sum_{ij} J_{ij} \boldsymbol{S}_i \cdot \boldsymbol{S}_j + \sum_{ijk\sigma\sigma'} K_{ijk} p_{i\sigma}^{\dagger} \big(\boldsymbol{S}_j \cdot \boldsymbol{\sigma}_{\sigma\sigma'} \big) p_{k\sigma'} \quad (7)$$

With

$$J_{ij} = \sum_{mm'} \frac{4\left|t_{ij}^{mm'}\right|^2}{9U_{\text{eff}}}, \; K_{ijk} = \sum_m \frac{4g_{ij}^m g_{kj}^{m*}}{3U_{\text{eff}}} \tag{8}$$

The obtained form is identical to that of the simplified version (Equation 2 of the main text) but with $S = 3/2$ rather than $S = 1/2$. The last term can be thought as the Kondo coupling between the local Cr spin $\boldsymbol{S}_j$ and neighbouring Pd electrons. Representative values are determined from the DFT calculations, and are listed in Table S2.

Table S2. Table of spin coupling constants derived from the strong coupling expansion.

$\boldsymbol{R}_j - \boldsymbol{R}_i$	$(\frac{3}{2}, \frac{\sqrt{3}}{2}, 0)$	$(-\frac{1}{2}, -\frac{\sqrt{3}}{2}, 1)$	$(-2, 0, 1)$
J_{ij} (meV)	5.17	0.166	0.106

$\boldsymbol{R}_j - \boldsymbol{R}_i$	$(-1, 0, \frac{1}{2})$	$(2, 0, \frac{1}{2})$	$(2, \sqrt{3}, \frac{1}{2})$
K_{iji} (meV)	3.39	6.71	1.19

These values of superexchange interactions J_{ij} between Cr $S = 3/2$ spins are in good agreement with those evaluated from the converged DFT+DMFT results with the linear-response theory of Ref. (*41*) for several first correlation shells (fig. S5). The obtained values are listed in Supplementary Table S3.

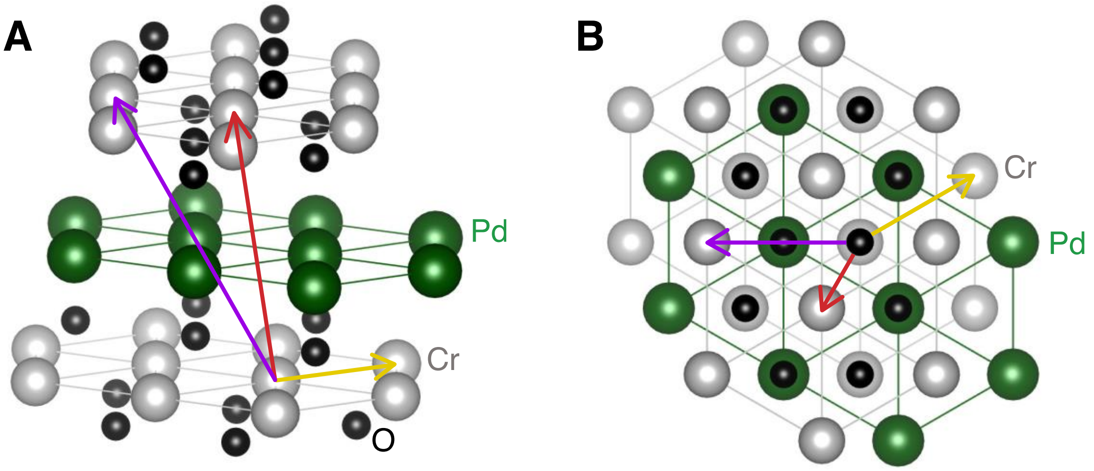

Fig. S5. Exchange pathways in $PdCrO_2$. A side view (**A**) and a view from above (**B**) of the crystal structure of $PdCrO_2$, with superexchange interactions marked (see Tables S2 and S3). Yellow, red and purple arrows indicate the lattice vectors $\boldsymbol{R}_i - \boldsymbol{R}_j = \left(\frac{3}{2}, \frac{\sqrt{3}}{2}, 0\right), \left(-\frac{1}{2}, -\frac{\sqrt{3}}{2}, 1\right)$ and $(-2,0,1)$ respectively.

3.2 Spectral function

The spectral function of Cr electrons is represented as

$$A_{\mathrm{Cr}}(\boldsymbol{k},\omega)=\sum_{m\sigma\alpha\alpha'}\left|\sum_i\langle\alpha_o^{(N)}|c_{im\sigma}|\alpha_o'^{(N+1)}\rangle e^{-i\boldsymbol{k}\boldsymbol{r}_i}\right|^2\delta\left(\omega+E_\alpha^{(N)}-E_{\alpha'}^{(N+1)}\right)\frac{e^{-\beta E_\alpha^{(N)}}}{Z}\left(1+e^{-\beta\omega}\right) \tag{9}$$

Table S3. Cr-Cr superexchange interactions calculated by the DFT + DMFT method. The lattice vectors for which the interactions J_{ij} are calculated are indicated in fig. S4.

$\boldsymbol{R}_j-\boldsymbol{R}_i$	$(\frac{3}{2},\frac{\sqrt{3}}{2},0)$	$(-\frac{1}{2},-\frac{\sqrt{3}}{2},1)$	$(-2,0,1)$
J_{ij} (meV)	6.24	−0.06	0.12

where $\left|\alpha_0^{(N)}\right\rangle$ is the eigenstate of the original Hamiltonian with N electrons and eigenenergy $E_\alpha^{(N)}$.We rewrite this expression in terms of the eigenstate of the effective Hamiltonian which is given as $|\alpha^{(N)}\rangle=e^{i\mathcal{S}}\left|\alpha_0^{(N)}\right\rangle$.

We can neglect terms with $E_\alpha^{(N)}=\mathcal{O}(U_{\mathrm{eff}})$ since $e^{-\beta E_\alpha^{(N)}}\ll 1$ holds for the temperature range of interest. Terms with $E_{\alpha'}^{(N+1)}=\mathcal{O}(U_{\mathrm{eff}})$ can also be neglected for the low-energy regime $|\omega|=\left|E_\alpha^{(N)}-E_{\alpha'}^{(N+1)}\right|\ll U_{\mathrm{eff}}$. Then the leading order expression in $1/U_{\mathrm{eff}}$ is obtained as

$$A_{\mathrm{Cr}}(\boldsymbol{k},\omega)\sim\sum_{m\sigma\alpha\alpha'}\left|\sum_i\langle\alpha^{(N)}|\left[i\mathcal{S}^{(1)},c_{im\sigma}\right]|\alpha'^{(N+1)}\rangle e^{-i\boldsymbol{k}\boldsymbol{r}_i}\right|^2\delta\left(\omega+E_\alpha^{(N)}-E_{\alpha'}^{(N+1)}\right)\frac{e^{-\beta E_\alpha^{(N)}}}{Z}\left(1+e^{-\beta\omega}\right) \tag{10}$$

$$\sim\sum_{m\sigma\alpha\alpha'}\left|\int\frac{d^3\boldsymbol{q}}{(2\pi)^3}\sum_{\sigma'}\frac{4g_{\boldsymbol{k}+\boldsymbol{q}}^{m*}}{3U_{\mathrm{eff}}}\langle\alpha^{(N)}|\boldsymbol{S}_{-\boldsymbol{q}}\cdot\boldsymbol{\sigma}_{\sigma\sigma'}p_{\boldsymbol{k}+\boldsymbol{q}\sigma'}|\alpha'^{(N+1)}\rangle\right|^2\delta\left(\omega+E_\alpha^{(N)}-E_{\alpha'}^{(N+1)}\right)\frac{e^{-\beta E_\alpha^{(N)}}}{Z}\left(1+e^{-\beta\omega}\right) \tag{11}$$

where $\boldsymbol{S}_{\boldsymbol{q}}=\sum_i\boldsymbol{S}_i\,e^{-i\boldsymbol{q}\cdot\boldsymbol{r}_i}$ and $g_{\boldsymbol{k}}^m=\sum_j g_{ij}^m\,e^{-i\boldsymbol{k}\cdot(\boldsymbol{r}_i-\boldsymbol{r}_j)}$. This expression can be equivalently obtained from the correlation function

$$G_{\mathrm{Cr}}^{\mathrm{eff}}(\boldsymbol{k},t)=-i\sum_{m\sigma\sigma'\sigma''}\int\frac{d^3\boldsymbol{q}}{(2\pi)^3}\int\frac{d^3\boldsymbol{q}'}{(2\pi)^3}\frac{16g_{\boldsymbol{k}+\boldsymbol{q}}^{m*}g_{\boldsymbol{k}+\boldsymbol{q}'}^{m}}{9U_{\mathrm{eff}}^2}\langle\left\{\boldsymbol{S}_{-\boldsymbol{q}}(t)\cdot\boldsymbol{\sigma}_{\sigma\sigma'}p_{\boldsymbol{k}+\boldsymbol{q}\sigma'}(t),\boldsymbol{S}_{\boldsymbol{q}'}\cdot\boldsymbol{\sigma}_{\sigma''\sigma}p_{\boldsymbol{k}+\boldsymbol{q}'\sigma''}^\dagger\right\}\rangle \tag{12}$$

with $O(t)$ being the Heisenberg representation (for the effective Hamiltonian) of an operator O. Namely, the low-energy excitation of the Cr electrons is described by a simultaneous disturbance on the metallic layer and the localized spin.
Since the coupling between the metallic and insulating layer is small ($\sim |g|^2/U_{\mathrm{eff}}$), we can decouple the expectation value $\langle SSpp^\dagger\rangle \sim \langle SS\rangle\,\langle pp^\dagger\rangle$ in the leading-order evaluation. This treatment is justified when the vertex correction (in terms of the diagrammatic expansion about the inter-layer coupling) can be neglected. The correlation function can then be approximated as

$$G_{\mathrm{Cr}}^{\mathrm{eff}}(\boldsymbol{k},t)\sim -i\sum_{m\sigma}\int\frac{d^3\boldsymbol{q}}{(2\pi)^3}\frac{16\left|g_{\boldsymbol{k}+\boldsymbol{q}}^m\right|^2}{9U_{\mathrm{eff}}^2}\left[\langle \boldsymbol{S}_{-\boldsymbol{q}}(t)\cdot\boldsymbol{S}_{\boldsymbol{q}}\rangle\langle p_{\boldsymbol{k}+\boldsymbol{q}\sigma}(t)p_{\boldsymbol{k}+\boldsymbol{q}\sigma}^\dagger\rangle+\langle \boldsymbol{S}_{\boldsymbol{q}}\cdot\boldsymbol{S}_{-\boldsymbol{q}}(t)\rangle\langle p_{\boldsymbol{k}+\boldsymbol{q}\sigma}^\dagger p_{\boldsymbol{k}+\boldsymbol{q}\sigma}(t)\rangle\right] \quad (13)$$

where we neglect the off-diagonal element $\langle p_{\boldsymbol{k}+\boldsymbol{q},\sigma}(t)p_{\boldsymbol{k}+\boldsymbol{q}',\sigma'}^\dagger\rangle$ which can exist in the ordered phase. Equation (3) in the main text is obtained by considering the zero temperature limit of the Fourier-transform of the expression for $G_{\mathrm{Cr}}^{\mathrm{eff}}(\boldsymbol{k},t)$.

When the spins acquire a long-range order, the spin correlation function $\langle \boldsymbol{S}_{\boldsymbol{q}}\cdot\boldsymbol{S}_{-\boldsymbol{q}}(\omega)\rangle = \int dt\langle \boldsymbol{S}_{\boldsymbol{q}}\cdot\boldsymbol{S}_{-\boldsymbol{q}}(t)\rangle e^{-i\omega t}$ has a divergent peak at $\omega=0$ and characteristic values of $\boldsymbol{q}$. For the present case, the experimentally determined magnetic order *(13, 14)* has a 120-degree structure, i.e.,

$$\langle \boldsymbol{S}_i\rangle = \mathrm{S}\begin{pmatrix}\sin(\boldsymbol{Q}+\boldsymbol{Z})\cdot\boldsymbol{r}_i\\ \cos\boldsymbol{Q}\cdot\boldsymbol{r}_i\\ 0\end{pmatrix} \quad (14)$$

with $\boldsymbol{Q}=(2\pi/3\,,2\pi/3\,,0)$ and $\boldsymbol{Z}=(0,0,\pi)$. With this magnetic order, we obtain the back-folded steep bands as shown in Fig. 3E of the main text.

The spectral intensity of the back-folded band with the momentum shift of $\pm\boldsymbol{Q}$ can be approximated as

$$I_{\mathrm{Cr}}^{\pm\boldsymbol{Q}}(\boldsymbol{k})=\sum_m\frac{8S^2|g_{\boldsymbol{k}\pm\boldsymbol{Q}}^m|^2}{9U_{\mathrm{eff}}^2} \quad (15)$$

(fits to the calculated Cr spectral function are plotted as a function of energy in Fig. 3A of the main text (solid line)). This form of the intensity is substantially different from that of the back-folded band of Pd electrons due to the modified potential.

If we consider a bilayer case for simplicity, the ‘band folding’ model Pd Hamiltonian, with the modified potential due to the 120-degree structure, can be written in a 2×2 form

$$H=\sum_{\boldsymbol{k}}\begin{pmatrix}p_{\boldsymbol{k}\uparrow}\\ p_{\boldsymbol{k}+\boldsymbol{Q}\downarrow}\end{pmatrix}^\dagger\begin{pmatrix}\epsilon_{\boldsymbol{k}} & \Delta\\ \Delta & \epsilon_{\boldsymbol{k}+\boldsymbol{Q}}\end{pmatrix}\begin{pmatrix}p_{\boldsymbol{k}\uparrow}\\ p_{\boldsymbol{k}+\boldsymbol{Q}\downarrow}\end{pmatrix}. \quad (16)$$

The spectral intensity of the back-folded band with the momentum shift of $\pm\boldsymbol{Q}$ is obtained as

$$I_{\mathrm{Pd}}(\boldsymbol{k}) = 1 - \frac{|\epsilon_{\boldsymbol{k}} - \epsilon_{\boldsymbol{k}+\boldsymbol{Q}}|}{\sqrt{(\epsilon_{\boldsymbol{k}} - \epsilon_{\boldsymbol{k}+\boldsymbol{Q}})^2 + 4\Delta^2}}, \quad (17)$$

which rapidly decays as $I_{\mathrm{Pd}}(\boldsymbol{k}) \sim 2\,|\Delta/(\epsilon_{\boldsymbol{k}} - \epsilon_{\boldsymbol{k}+\boldsymbol{Q}})|^2$ as $|\epsilon_{\boldsymbol{k}} - \epsilon_{\boldsymbol{k}+\boldsymbol{Q}}|$ becomes large. We plot this form of intensity in Fig. 3A of the main text (dashed line), with $\Delta = 20$ meV, consistent with the experimental gap $2\Delta \sim 40$ meV estimated from the breakdown field reported in Ref. 16 as $\hbar\omega_c \sim \Delta^2/E_F$. We stress that, for such a "conventional" back-folding, the rapid suppression of spectral weight away from the magnetic Brillouin zone boundary is therefore intrinsic. In contrast, in the intertwined spin-charge model, the reconstructed weight in the Cr spectral function is approximately constant, varying only due to momentum-dependent variations of the inter-layer coupling term $g_{\boldsymbol{k}+\boldsymbol{Q}}$. Our calculations (fig. S6) show how this is sensitive to details of the calculation. Including only nearest-neighbour coupling, the spectral weight slightly decreases towards the Fermi level. Including next nearest-neighbour coupling, the spectral weight increases towards the Fermi level. In all cases, the variation in spectral weight is less than a factor of 2 over an energy range of more than 700 meV below the Fermi level, entirely consistent with our experimental measurements shown in Fig. 3A of the main text and Fig. S6. Throughout, we have shown the relative intensity variations between different models and the experimental data by normalising to the intensity at -0.7 eV binding energy; equivalent conclusions are drawn if normalising directly by the main band intensity, as shown in Fig. S7.

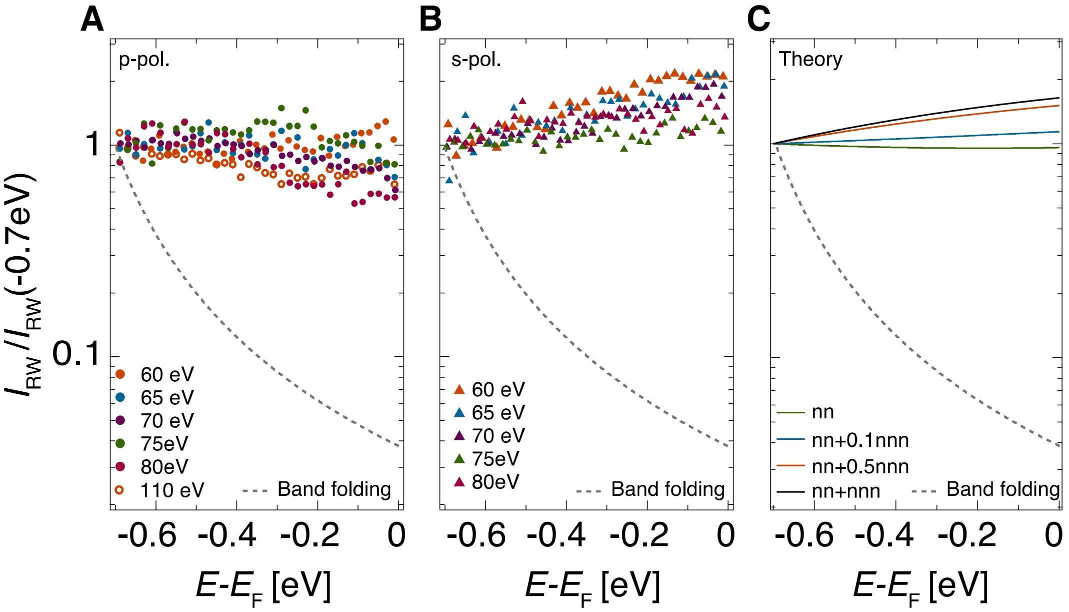

Fig. S6. Binding energy–dependent spectral weight. Reconstructed weight (I_{RW}) as obtained from fits to dispersions measured using various photon energies and (**A**) p-polarised and (**B**) s-polarised light. Although varying ARPES matrix elements cause some changes in the binding energy dependence of the reconstructed weight intensity for the different measurement conditions, the measured spectral weight never varies by more than a factor of two over the 700meV energy range. This is in sharp contrast to the prediction of the simple 'band folding' model. While binding energy independent 'shadow features' have previously been observed in materials with superperiodic structures or structural distortions (*42*), such underlying origins would be inconsistent with the Cr character of the reconstructed weight that we observe. (**C**) Cr I_{RW} predicted by the intertwined spin-charge model for various combinations of nearest neighbour (nn) and next-nearest neighbour (nnn) hopping parameters. Small parameter-dependent quantitative variations are observed, although the overall binding energy dependence of the reconstructed weight intensity remains weak for all parameters, again in clear contrast to the 'band folding' model.

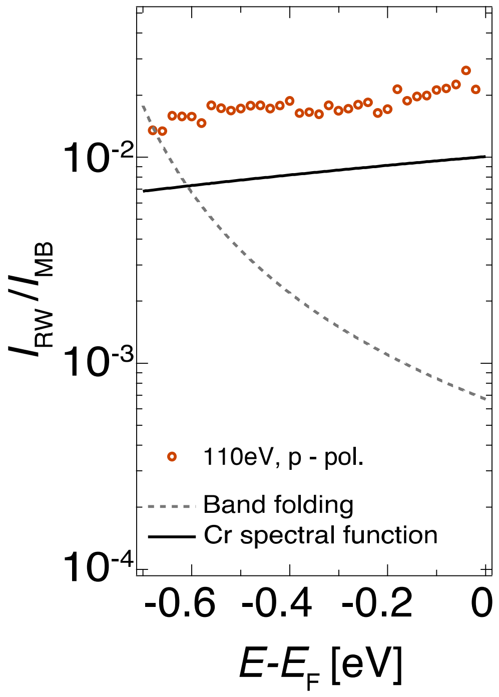

Fig. S7. The ratio of the reconstructed weight and the main band weight. The experimental results (symbols) are compared with the prediction of the band folding model (dashed line) and the Cr spectral function calculated by our theory (full line). Experimental data are divided by the calculated ratio of photoemission cross-sections of Cr 3d and Pd 4d orbitals, which is 11 at 110eV.
